# Integrative Evidence on Sesame Supplementation for Cardiometabolic Risk Factors Relevant to Retinopathy

**DOI:** 10.7150/ijms.123717

**Published:** 2026-01-23

**Authors:** Wu-Hsien Kuo, Ko-Shih Chang, Fu-Hsuan Kuo, Kuan-Po Cheng, Ru-Yin Tsai

**Affiliations:** 1 Department of Gastroenterology and Hepatology, Yuan Sheng Branch, Yuan Rung Hospital, Yuanlin, Changhua, 510007, Taiwan.; 2 Department of Cardiology, Yuan Rung Hospital, Yuanlin, Changhua, 510005, Taiwan.; 3 Center for Geriatrics & Gerontology, Taichung Veterans General Hospital, Taichung 407219, Taiwan.; 4 Division of Neurology, Taichung Veterans General Hospital, Taichung 407219, Taiwan.; 5 School of Medicine, Chung Shan Medical University, Taichung 40201, Taiwan.; 6 Department of Anatomy, School of Medicine, Chung Shan Medical University, Taichung 40201, Taiwan.; 7 Department of Medical Education, Chung Shan Medical University Hospital, Taichung 40201, Taiwan.

**Keywords:** inflammation, non-alcoholic fatty liver, lipid, type 2 diabetes, dyslipidemia, obesity, insulin

## Abstract

**Background:**

Cardiometabolic disorders, such as diabetes, hypertension, dyslipidemia, retinopathy, and non-alcoholic fatty liver disease, present significant health challenges globally. Recent evidence suggests that sesame (*Sesamum indicum L.*) supplementation may offer beneficial effects in modulating various cardiometabolic risk factors, although findings from clinical trials have been inconsistent.

**Objective:**

This meta-analysis aims to systematically assess the effects of sesame supplementation on multiple cardiometabolic parameters, including lipid profiles, blood pressure, glycemic control, liver enzyme levels, inflammatory biomarkers, body weight, and body mass index (BMI), with the goal of evaluating its potential as an adjunctive therapy for clinical retinopathy.

**Methods:**

A comprehensive literature search was conducted across multiple databases through July 2025 to identify randomized controlled trials (RCTs) that compared sesame supplementation with placebo or active controls on cardiometabolic outcomes.

**Results:**

Pooled effect sizes were calculated using a random-effects model. A total of 10 studies involving 441 participants were included in the meta-analysis. Sesame supplementation significantly reduced both systolic and diastolic blood pressure. Improvements were also observed in glycemic control, with reductions in fasting blood glucose and HbA1c levels. Further-more, sesame intake was associated with a significant reduction in liver enzyme levels, particularly alanine aminotransferase (ALT). Subgroup analyses revealed that the effects did not increase with longer durations of sesame supplementation.

**Conclusions:**

This meta-analysis provides evidence supporting the beneficial effects of sesame supplementation in improving various cardiometabolic risk factors. Incorporating sesame products into dietary strategies may offer a promising adjunctive intervention for managing cardiometabolic disorders and retinopathy associated with these disorders.

## Introduction

Cardiometabolic diseases, encompassing conditions such as hypertension, dyslipidemia, type 2 diabetes mellitus, obesity, retinopathy, and non-alcoholic fatty liver disease, remain leading contributors to global morbidity and mortality 1. These disorders often coexist and share underlying pathophysiological mechanisms, including insulin resistance, chronic low-grade inflammation, oxidative stress, and dysregulated lipid metabolism 2, 3. The escalating prevalence of these conditions, particularly in low- and middle-income countries, underscores the urgent need for effective and sustainable preventive and therapeutic strategies 4, 5.

Dietary approaches are considered a key component in the prevention and management of cardiometabolic risk factors. In recent years, growing attention has been given to the potential benefits of functional foods and plant-based compounds 6. *Sesamum indicum L.*, commonly known as sesame, is a traditional oilseed that has been used in human diets for centuries. It contains several bioactive components, including lignans (such as sesamin), unsaturated fatty acids, tocopherols, and phytosterols, which have demonstrated antioxidant, anti-inflammatory, lipid-lowering, and antihypertensive effects in experimental models 7.

In addition to its effects on traditional cardiometabolic risk factors, emerging findings from animal and cellular models suggest that sesame supplementation may exert biological effects relevant to retinopathy, a common microvascular complication of metabolic disorders, particularly diabetes 8, 9. Retinopathy, which can lead to vision impairment and blindness, shares pathophysiological links with cardiometabolic diseases, including oxidative stress, inflammation, and impaired vascular function 2, 7, 10. Given the bioactive compounds in sesame, such as lignans and tocopherols, which possess antioxidative and anti-inflammatory properties 11, sesame may serve as an adjunctive therapeutic approach for managing retinopathy in patients with underlying cardiometabolic conditions. However, the evidence on the effects of sesame supplementation on retinopathy remains limited and warrants further investigation.

Although clinical studies have explored the health effects of sesame supplementation, the findings have been inconsistent, and the overall impact on cardiometabolic outcomes remains unclear 12, 13. Some trials have reported improvements in blood pressure, glycemic control, lipid profiles, and liver enzymes, while others have shown limited or no significant effects 13-16. A comprehensive synthesis of the available evidence is therefore needed to better understand the role of sesame in cardiometabolic risk factors and to provide a basis for considering sesame as an adjunctive therapy for clinical retinopathy.

## Materials and Methods

### Data sources and selection criteria

This meta-analysis systematically identified RCTs assessing the impact of sesame supplementation in individuals with metabolic disorders. A comprehensive literature search was carried out using PubMed, Embase, the Cochrane Library, and Web of Science for studies published up to February 2025. Search terms combined phrases such as "Sesamum"[Mesh] OR "sesame" OR "sesame oil" OR "sesame seed" OR "sesame lignan") AND ("Cardiometabolic Risk"[Mesh] OR "Metabolic Syndrome"[Mesh] OR "Dyslipidemias"[Mesh] OR "Insulin Resistance"[Mesh] OR "Hypertension"[Mesh] OR "Blood Glucose"[Mesh] OR "Triglycerides"[Mesh] OR "Cholesterol"[Mesh] OR "Body Mass Index"[Mesh] OR "HDL" OR "LDL" OR "glycemic control" OR "lipid profile") AND ("Retinopathy"[Mesh] OR "Diabetic Retinopathy"[Mesh] OR "hypertensive retinopathy" OR "retinal vascular disease" OR "retinal microvasculature" with an emphasis on human clinical research. The review process adhered to the PRISMA guidelines. Additionally, reference lists of all included studies were manually screened to capture any further eligible trials. Studies such as case reports, technical documents, abstracts from conferences, reviews, editorials, letters, and non-clinical research were excluded. The protocol for this review was registered in PROSPERO with the registration number CRD 42024603099.

### Selection of studies

**Figure 1:**
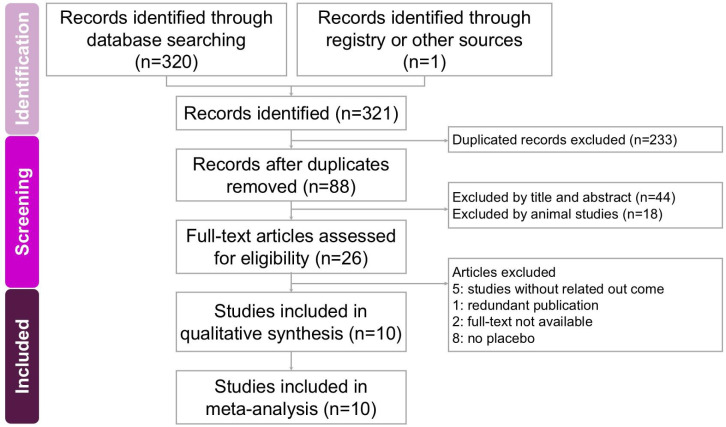
** A flowchart illustrating the study selection process for the systematic review and meta-analysis on the effects of sesame supplementation in managing cardiometabolic risk factors.** Of the 321 records initially identified, 10 studies met the eligibility criteria and were included in the final analysis.

Two researchers independently conducted the screening and assessment of eligible studies, with a third researcher providing oversight to maintain accuracy and consistency throughout the process. All relevant articles were obtained in full text and carefully examined to ensure thorough evaluation. The step-by-step selection procedure is illustrated in the PRISMA flow diagram (Figure 1).

### Data extraction

Two researchers independently extracted data using a standardized form based on the Cochrane Handbook recommendations 17. The extracted information included the authors and year of publication, study location, participant eligibility criteria, demographic characteristics such as sample size and age range, study design, intervention details, measured outcomes, and the assessment methods used.

### Outcomes

The primary outcomes assessed in this study included systolic and diastolic blood pressure, fasting blood glucose, body weight, and body mass index. Secondary outcomes encompassed liver enzymes (ALT and AST), lipid profiles (total cholesterol, triglycerides, HDL, and LDL), fasting plasma insulin, HbA1c, HOMA-IR, and inflammatory markers such as TNF-α, hs-CRP, and IL-6.

### Assessment of methodological quality

Two independent researchers carefully assessed the risk of bias in the included studies using the Cochrane Collaboration's Risk of Bias tool to evaluate methodological quality. Any differences in their evaluations were resolved through discussion with a third reviewer to reach agreement. Studies were classified as having a high risk of bias if concerns were noted in one or more of the tool's assessment domains.

### Data analysis

Quantitative synthesis was conducted using Standardized Mean Differences (SMDs) with corresponding 95% Confidence Intervals (CIs) to compare outcomes between the intervention and control groups. A random-effects model was applied to account for variability across studies. All statistical analyses were performed using Comprehensive Meta-Analysis software, version 3 (Biostat, Englewood, NJ, USA). Heterogeneity was evaluated using the *I²* statistic, with values above 50% considered indicative of substantial heterogeneity. Publication bias was assessed through funnel plot inspection and Egger's regression test, applying a significance level of *p* < 0.05 for most outcomes and *p* < 0.10 for bias detection. Subgroup analyses were conducted to explore potential sources of heterogeneity, and sensitivity analyses were carried out by sequentially removing individual studies to test the robustness of the findings.

## Results

### Study search and characteristics of included patients

The process of study screening and selection is presented in Figure 1. Initially, a systematic search conducted in four databases (PubMed, Embase, Cochrane Library, and Web of Science) identified 321 potentially relevant studies. After duplicates were removed, 88 articles underwent title and abstract screening, resulting in 62 exclusions. The remaining 26 studies were further evaluated through comprehensive full-text assessments, leading to the exclusion of 16 trials due to unrelated outcomes (5 studies 18-22), duplicate publications (1 study 23), lack of accessible full texts (2 studies 24, 25), and absence of a placebo-controlled group (8 studies 26-33). Ultimately, 10 RCTs published in English between 2009 and 2022 were included in the meta-analysis, comprising a total of 441 participants 12-16, 34-38. These studies primarily investigated the effects of sesame-based interventions on liver fat content and lipid profiles. Key characteristics of the included studies are summarized in Table 1.

### Quality assessment

**Figure 2:**
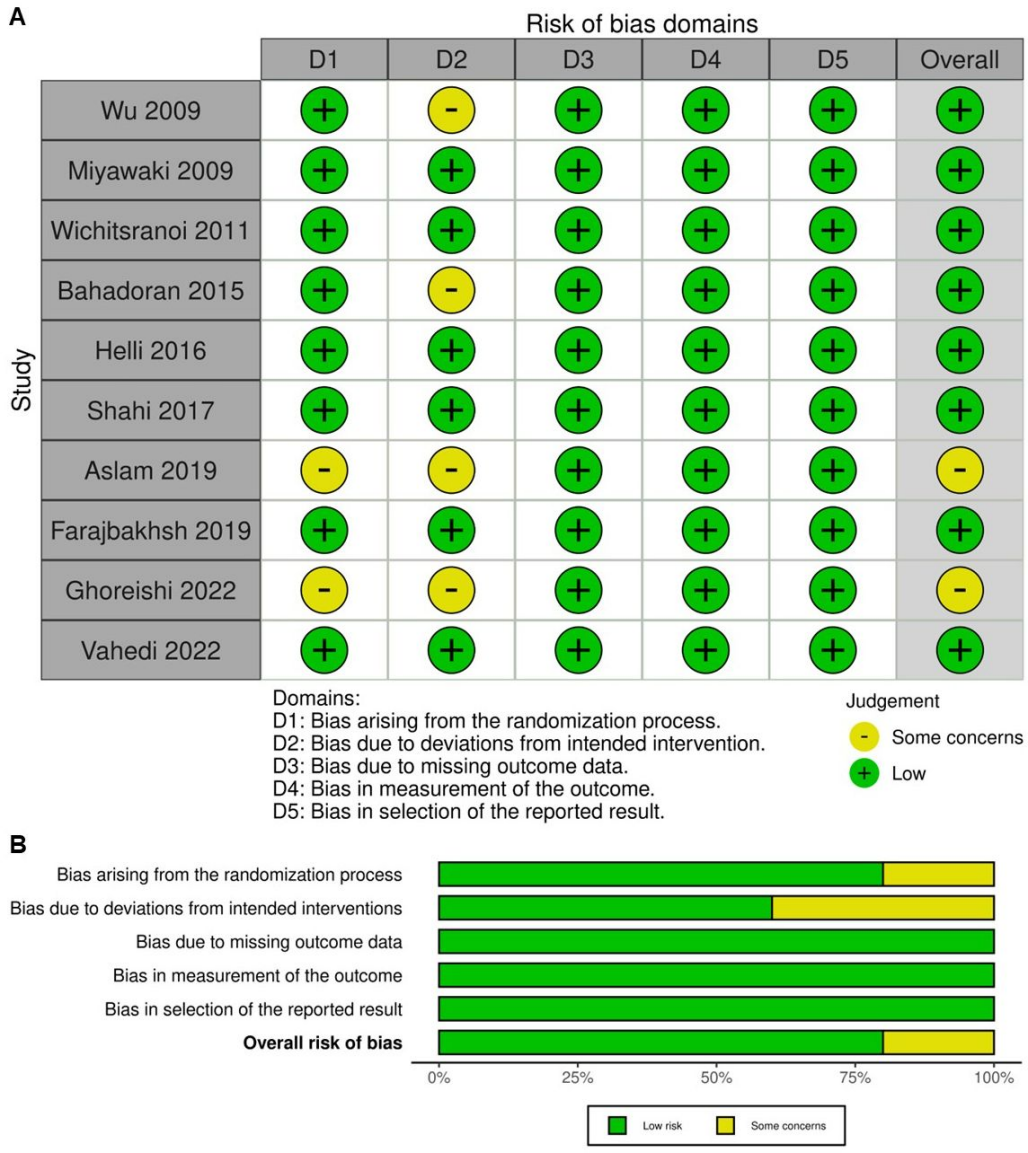
** Evaluation of the methodological quality of the included trials.** (A) Individual risk of bias assessment for each selected study, based on the Rob 2.0 tool (https://mcguinlu.shinyapps.io/robvis/). (B) Overall risk of bias summarized as a percentage, considering intention-to-treat and perprotocol analyses. The primary sources of high risk of bias across the studies were deviations from intended interventions, followed by issues related to missing outcome data and deficiencies in the randomization process.

The methodological quality of the ten included studies was evaluated using the Cochrane Collaboration's Risk of Bias 2 tool. Most trials demonstrated a generally high methodological standard, with randomization methods and allocation concealment clearly described and baseline comparability established in nearly all cases. Biases arising from the randomization process were minimal, reflecting the strengths of RCT design. However, some concerns were identified in several studies, particularly relating to deviations from intended interventions. This was most commonly due to practical limitations in blinding participants to the intervention, especially in studies involving visible dietary changes (e.g., sesame oil versus control oils, or sesame-containing foods). Despite double-blind or single-blind designs being used in most trials, true participant blinding was not always feasible, leading to potential performance bias. Missing outcome data were generally infrequent, and attrition was low across studies, with reasons for withdrawal typically unrelated to intervention or outcome. Outcome measurements were based on objective laboratory or clinical parameters and were assessed with standardized protocols, reducing the risk of detection bias. Selective reporting bias was also low, with most studies reporting all pre-specified outcomes according to their analysis plans. These assessments are visually represented in Figure 2, with Figure 2A displaying each study's risk of bias across all domains and Figure 2B summarizing the proportion of studies at each risk level within each domain. In summary, the included RCTs were of reasonable methodological rigor, with minimal risk of bias in randomization and outcome measurement.

### Effect of sesame on blood pressure

**Figure 3:**
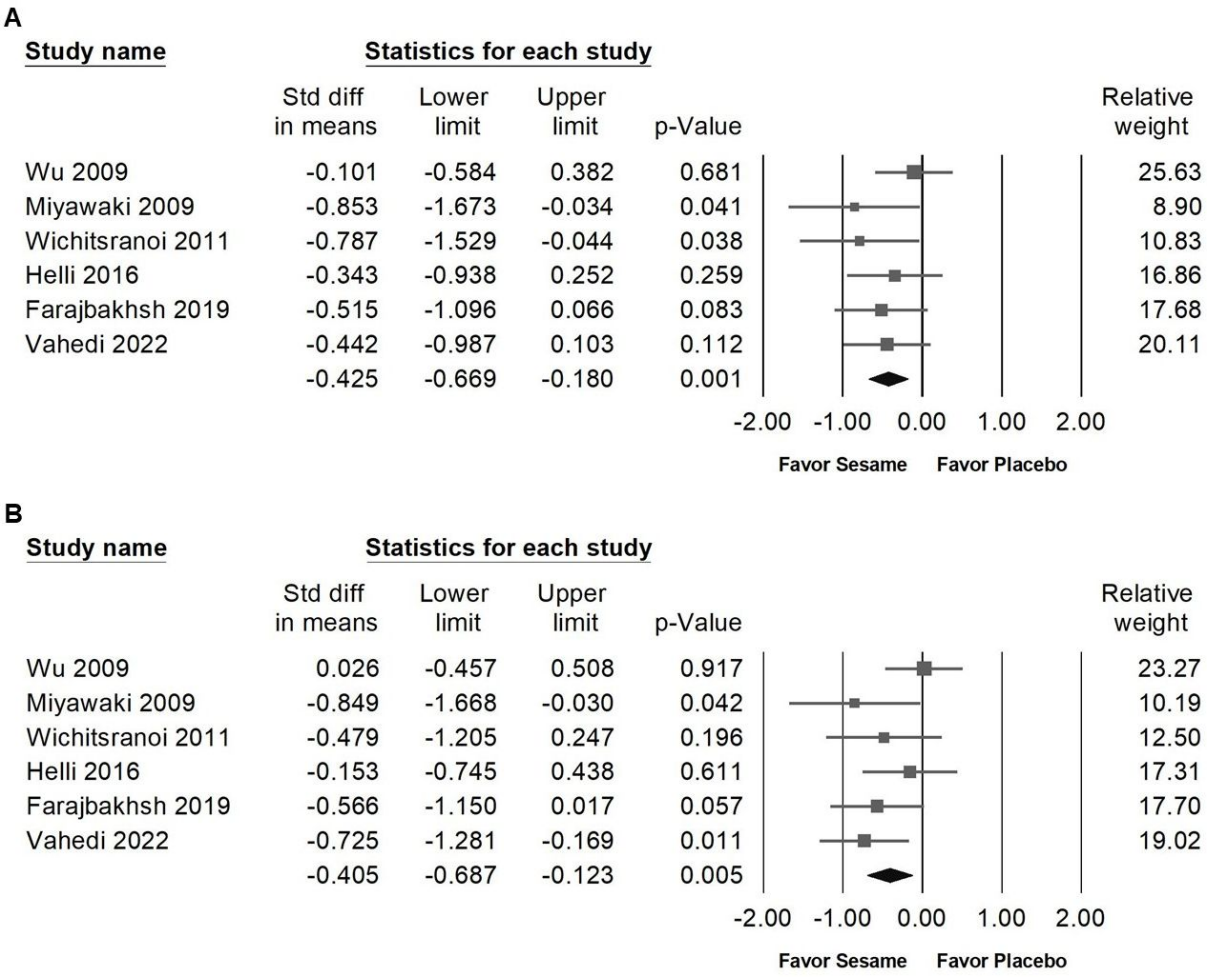
** Forest plots showing the effects of sesame supplementation on blood pressure.** Panel (A) illustrates the effects on systolic blood pressure, and panel (B) presents the effects on diastolic blood pressure. Individual studies are represented by squares, with horizontal lines indicating 95% confidence intervals. Diamonds at the bottom represent the pooled effect sizes for each blood pressure outcome.

**Figure 4:**
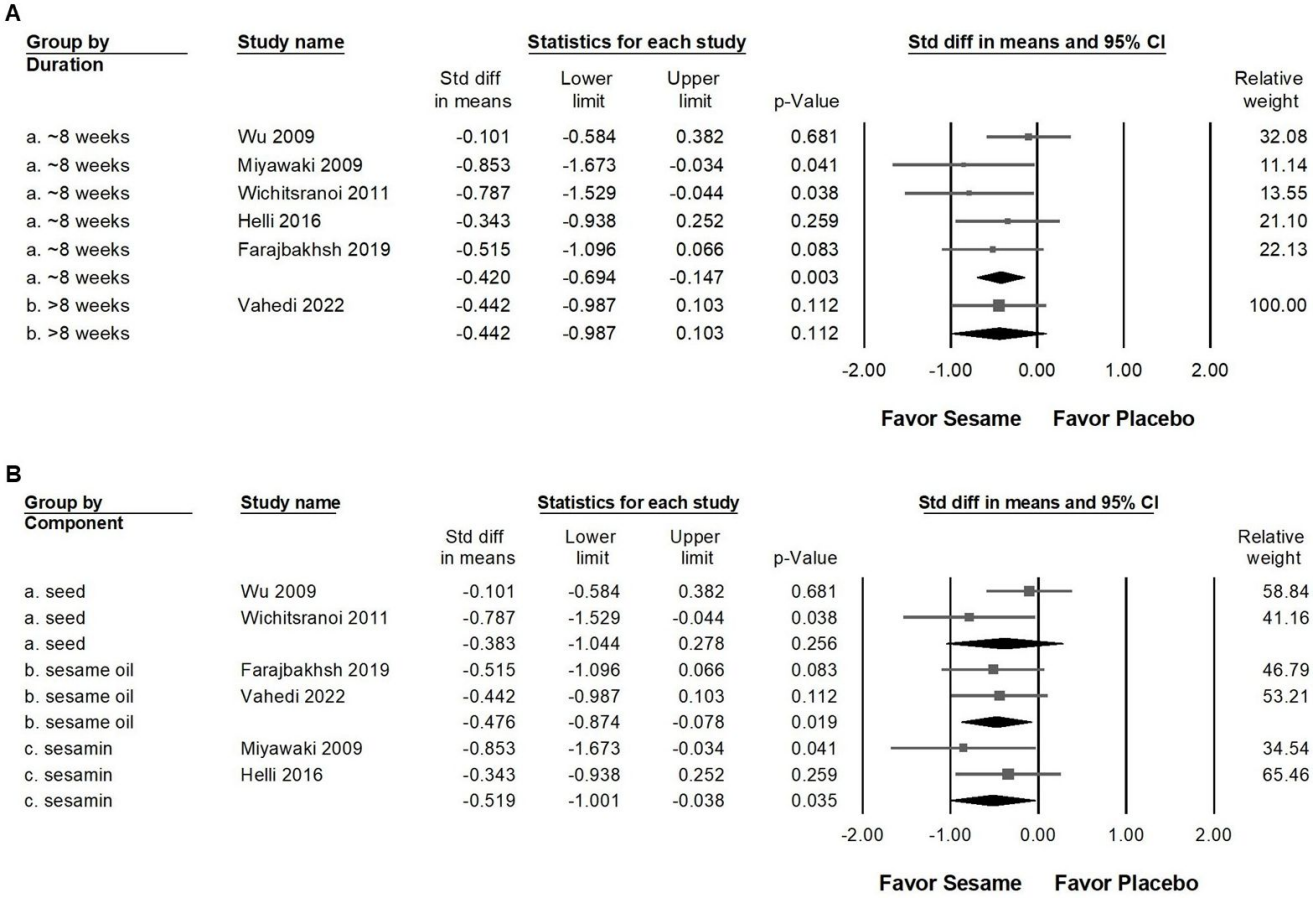
** Subgroup analyses corresponding to the effects presented in Figure 3A.** Panel (A) assesses differences according to the duration of sesame supplementation, and Panel (B) evaluates effects based on sesame component type. Individual effect sizes are shown as squares, with their position indicating the standardized mean difference; squares positioned to the left indicate a reduction in blood pressure. Horizontal lines represent the 95% confidence intervals, and the diamond at the bottom illustrates the pooled effect size for each subgroup analysis.

Sesame supplementation demonstrated a small yet statistically significant reduction in systolic blood pressure (Figure 3A, SMD -0.425, 95% CI -0.669 to -0.180, *p* = 0.001; *I²* < 0.001%, *p* = 0.571) and diastolic blood pressure (Figure 3B, SMD -0.405, 95% CI -0.687 to -0.123, *p* = 0.005; *I²* = 22.784%, *p* = 0.263). In subgroup analyses stratified by intervention duration (Figure 4A), sesame supplementation showed a small but significant effect on systolic blood pressure in studies lasting ≤8 weeks (SMD -0.420, 95% CI -0.694 to -0.147, *p* = 0.003; *I²* < 0.001%, *p* = 0.427), whereas no significant effect was observed in studies exceeding 8 weeks (SMD -0.442, 95% CI -0.987 to 0.103, *p* = 0.112;* I²* < 0.001%, *p* > 0.999). When evaluating the different types of sesame preparations (Figure 4B), sesamin exhibited a moderate effect on lowering systolic blood pressure (SMD -0.519, 95% CI -1.001 to -0.038, *p* = 0.035; *I²* < 0.001%, *p* = 0.324). Sesame oil supplementation resulted in a smaller but still significant reduction (SMD -0.476, 95% CI -0.874 to -0.078, *p* = 0.019; *I²* < 0.001%, *p* = 0.858). Conversely, sesame seeds showed no statistically significant impact (SMD -0.383, 95% CI -1.044 to 0.278, *p* = 0.256; *I²* = 56.479%, *p* = 0.130).

### Impact of sesame supplementation on fasting blood glucose, fasting serum insulin, HbA1c, and HOMA-IR

**Figure 5:**
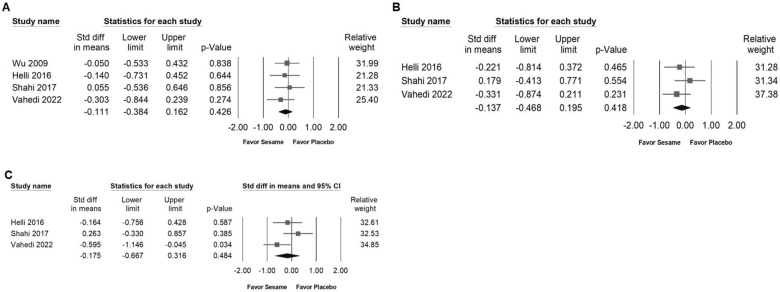
** Forest plots depicting the impact of sesame supplementation on glycemic control outcomes.** Panel (A) presents the effects on fasting blood glucose, Panel (B) fasting insulin, Panel (C) HbA1c levels, and Panel (D) HOMA-IR. Each square represents the standardized mean difference of individual studies, accompanied by horizontal lines indicating the 95% confidence intervals. The diamond at the base of each panel shows the pooled effect size, summarizing the overall effect of sesame supplementation on each glycemic marker.

Sesame supplementation was associated with a modest but significant reduction in fasting blood glucose levels (SMD -0.300, 95% CI -0.560 to -0.040; *I²* < 0.001%, *p* = 0.531; Figure 5A) and exhibited a moderate effect on HbA1c (SMD -0.658, 95% CI -1.163 to -0.154; *I²* = 56.273%, *p* = 0.102; Figure 5C). However, sesame had no significant impact on fasting serum insulin levels (SMD -0.125, 95% CI -0.463 to 0.214; *I²* = 20.494%, *p* = 0.287; Figure 5B) or on HOMA-IR values (SMD -0.019, 95% CI -0.368 to 0.329; *I²* < 0.001%, *p* = 0.867; Figure 5D).

### Impact of sesame on body weight, BMI, body fat, waist circumference, and hip circumference

**Figure 6:**
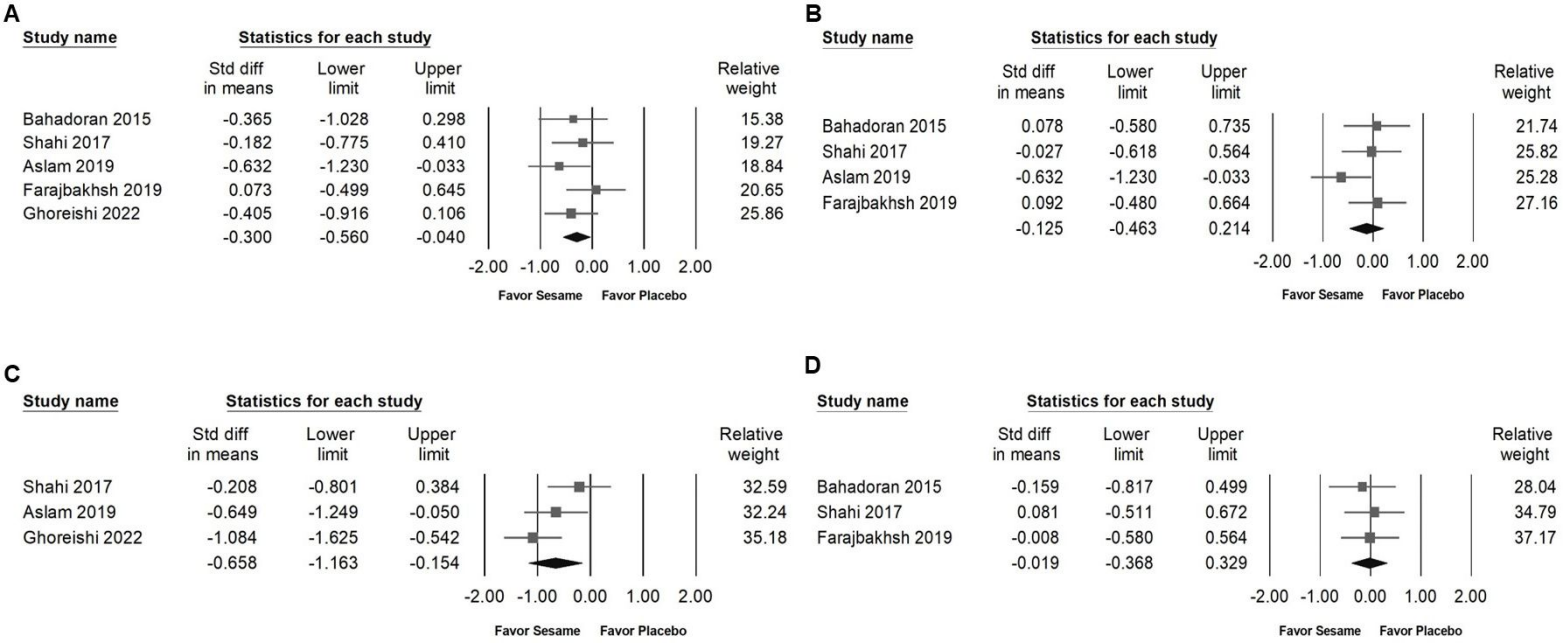
** Forest plots demonstrating the effects of sesame supplementation on anthropometric outcomes.** Panel (A) illustrates the impact on body weight, Panel (B) shows the effect on body mass index (BMI), and Panel (C) presents the changes in body fat percentage. Individual effect sizes are represented by squares, with horizontal lines indicating the 95% confidence intervals. The diamonds at the bottom of each panel represent the pooled effect sizes, summarizing the overall influence of sesame supplementation on these anthropometric measures.

**Figure 7:**
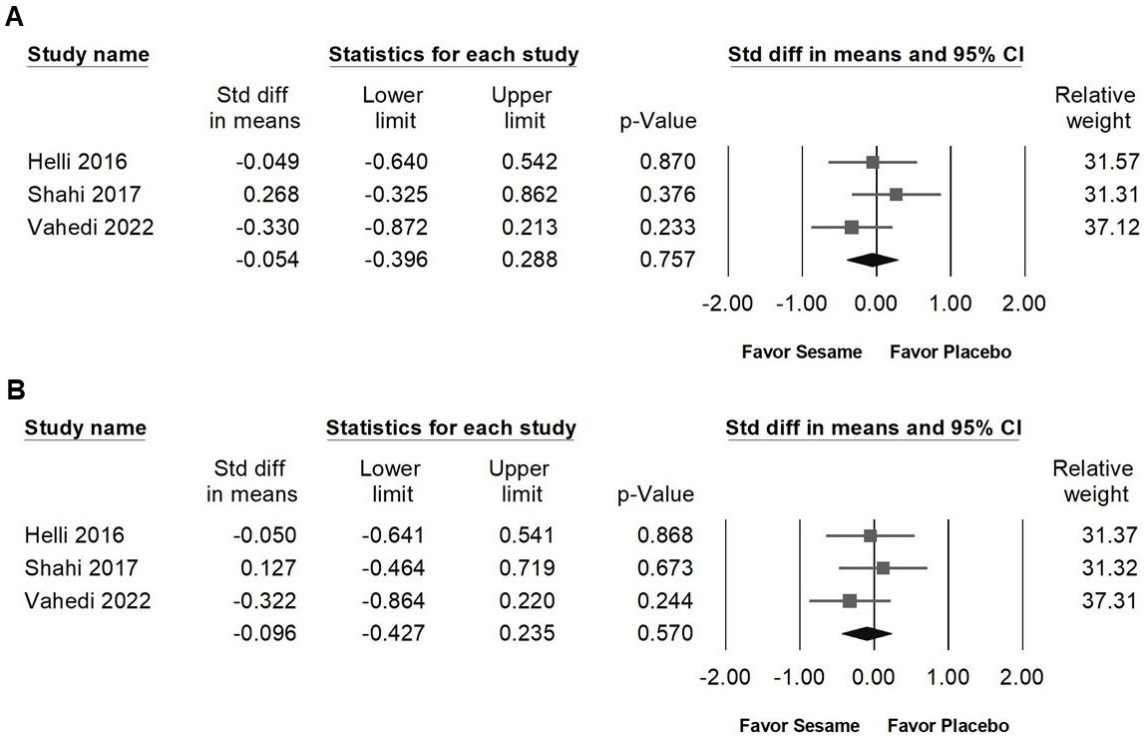
** Forest plots illustrating the impact of sesame supplementation on waist and hip circumference.** Panel (A) shows changes in waist circumference, while Panel (B) depicts changes in hip circumference. Squares represent individual study effect sizes, with horizontal lines indicating their respective 95% confidence intervals. The diamonds at the bottom summarize the pooled effect sizes, reflecting the overall effects of sesame supplementation on these anthropometric outcomes.

Sesame supplementation showed no significant effects on body weight (SMD -0.111, 95% CI -0.384 to 0.162; *I²* < 0.001%, *p* = 0.837; Figure 6A), BMI (SMD -0.137, 95% CI -0.468 to 0.195; *I²* < 0.001%, *p* = 0.436; Figure 6B), body fat percentage (SMD -0.175, 95% CI -0.667 to 0.316; *I²* = 53.803%, *p* = 0.115; Figure 6C), waist circumference (SMD -0.054, 95% CI -0.396 to 0.288; *I²* = 5.879%, *p* = 0.346; Figure 7A), or hip circumference (SMD -0.096, 95% CI -0.427 to 0.235; *I²* < 0.001%, *p* = 0.538; Figure 7B).

### Influence of sesame on blood lipids, lipoproteins, and liver injury markers

**Figure 8:**
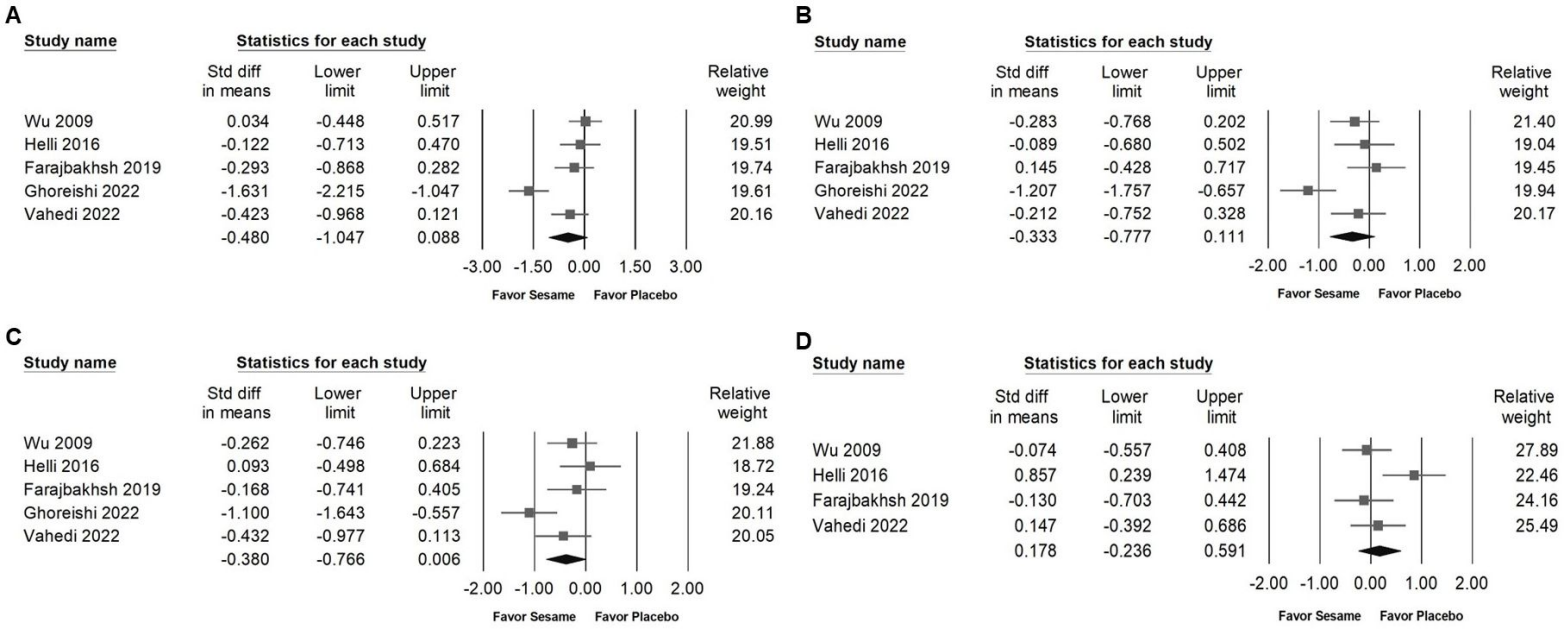
** Forest plots presenting the effects of sesame supplementation on lipid profile parameters.** Panel (A) shows the impact on total cholesterol, panel (B) illustrates changes in triglyceride levels, panel (C) depicts effects on LDL cholesterol, and panel (D) examines alterations in HDL cholesterol. Individual study effects are represented by squares, with horizontal lines indicating the corresponding 95% confidence intervals. The diamonds at the bottom of each panel represent the pooled effect sizes, summarizing the overall impact of sesame supplementation on these lipid outcomes.

**Figure 9:**
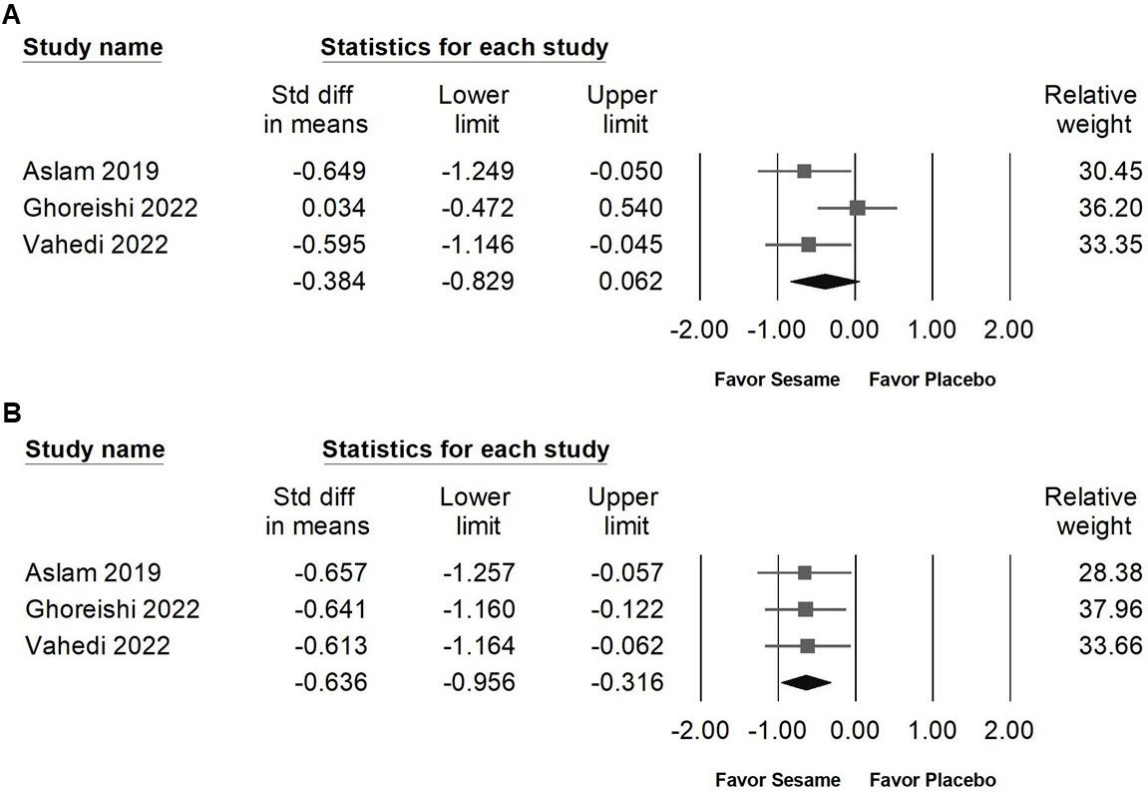
** Forest plots demonstrating the effects of sesame supplementation on liver injury markers.** Panel (A) presents the effects on AST levels, and panel (B) shows changes in ALT levels. Individual study results are indicated by squares, accompanied by horizontal lines denoting the 95% confidence intervals. Diamonds at the bottom of each panel summarize the overall pooled effect sizes, representing the cumulative impact of sesame supplementation on liver function parameters.

Sesame supplementation did not significantly affect serum cholesterol levels (SMD -0.480, 95% CI -1.047 to 0.088; *I²* = 80.983%, *p* < 0.001; Figure 8A), triglycerides (SMD -0.333, 95% CI -0.777 to 0.111; *I²* = 69.801%, *p* < 0.001; Figure 8B), LDL cholesterol (SMD -0.380, 95% CI -0.766 to 0.006; *I²* = 59.978%, *p* = 0.041; Figure 8C), or HDL cholesterol (SMD 0.178, 95% CI -0.236 to 0.591; *I²* = 55.758%, *p* = 0.079; Figure 8D). Regarding liver function indicators, sesame supplementation showed a moderate and significant reduction in ALT levels (SMD -0.636, 95% CI -0.956 to -0.316; *I²* < 0.001%, *p* = 0.994; Figure 9B), whereas no significant effect was observed on AST levels (SMD -0.384, 95% CI -0.829 to 0.062; *I²* = 49.039%, *p* = 0.141; Figure 9A). These results indicate that sesame supplementation may selectively improve certain aspects of liver function but has limited effects on lipid profiles.

### Influence of sesame on inflammatory markers

**Figure 10:**
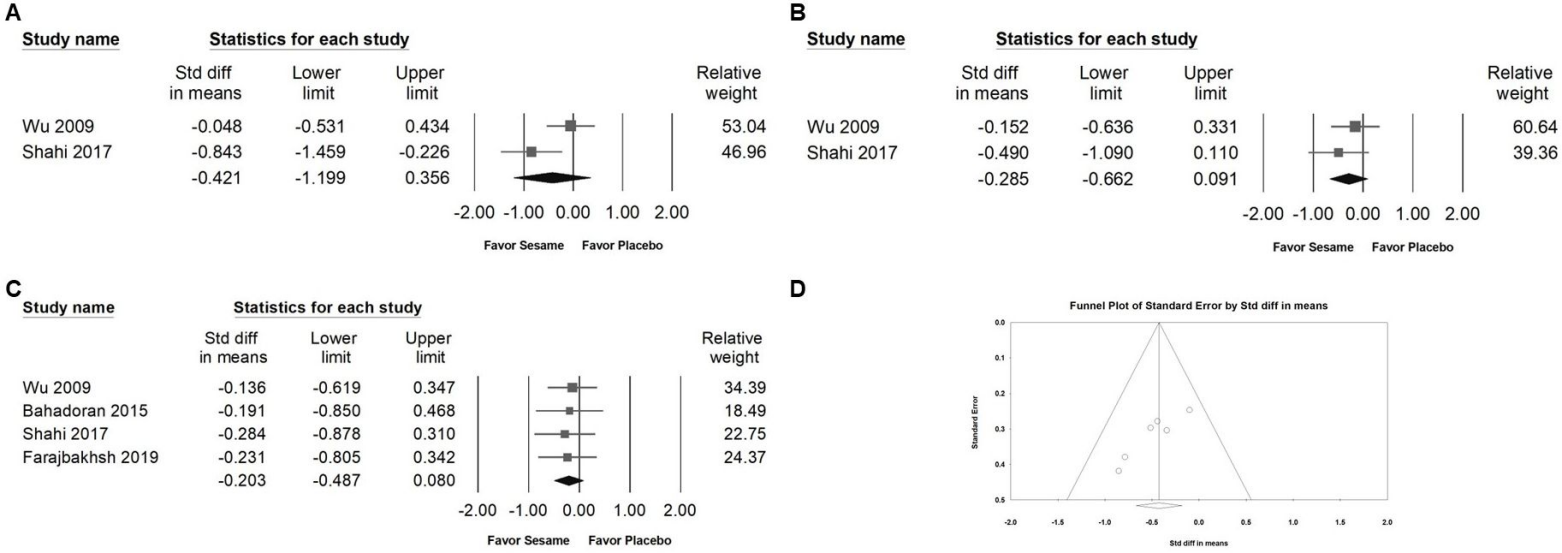
** Forest plots illustrating the effects of sesame supplementation on inflammatory markers.** Panel (A) presents the effects on TNF-α levels, Panel (B) depicts changes in IL-6, and Panel (C) summarizes findings for hs-CRP. Squares indicate the standardized mean differences for individual studies, with horizontal lines representing the 95% confidence intervals. Diamonds at the bottom of each panel reflect the overall pooled effect sizes. Panel (D) displays a funnel plot assessing publication bias among studies evaluating systolic blood pressure (as shown in Figure 3A). Each circle represents an individual study, with circle size corresponding to the study's relative weight or sample size. The lines denote confidence intervals around the pooled effect size, while the central diamond illustrates the overall effect estimate and its 95% confidence interval.

Sesame supplementation did not significantly influence the levels of pro-inflammatory cytokines, including TNF-α (SMD -0.421, 95% CI -1.199 to 0.356; *I²* = 74.716%, *p* = 0.047; Figure 10A), IL-6 (SMD -0.285, 95% CI -0.662 to 0.091; *I²* < 0.001%, *p* = 0.390; Figure 10B), or hs-CRP (SMD -0.203, 95% CI -0.487 to 0.080; *I²* < 0.001%, *p* = 0.984; Figure 10C). These results suggest that sesame supplementation has minimal or no impact on inflammatory markers associated with cardiometabolic risk.

### Publishing bias

Egger's regression analysis revealed significant publication bias in the dataset (*p* = 0.00862; Figure 10D). Funnel plots illustrating the standardized mean differences for the effects of sesame supplementation on systolic blood pressure levels are presented in Figure 3A.

## Discussion

This meta-analysis provides an updated and comprehensive evaluation of the effects of sesame supplementation on a wide range of cardiometabolic risk factors, including blood pressure, glycemic indicators, anthropometric measures, liver enzymes, inflammatory markers, and retinopathy. The findings indicate that sesame supplementation yields modest but statistically significant improvements in systolic and diastolic blood pressure, fasting blood glucose, HbA1c, and ALT levels. In contrast, no significant effects were observed for outcomes such as body weight, BMI, body fat percentage, lipid parameters, AST, or inflammatory cytokines. In comparison to previous meta-analyses 7, 39 which primarily emphasized lipid and glycemic outcomes, the present study expands the scope by incorporating additional clinical endpoints, such as liver function, inflammation, and liver injury enzymes. It also provides more detailed subgroup analyses that examine the effects of different forms of sesame, including seeds, oil, and sesamin extract, as well as the influence of intervention duration. The analysis shows that shorter interventions, lasting eight weeks or less, are more likely to result in noticeable reductions in blood pressure, suggesting that sesame supplementation may have a quick effect on blood pressure. By incorporating recent studies published through early 2025, this review updates and broadens the available evidence on the clinical potential of sesame supplementation, particularly in managing cardiometabolic disorders and retinopathy associated with cardiometabolic risk factors.

Our meta-analysis demonstrated that sesame supplementation modestly reduces both systolic and diastolic blood pressure. This antihypertensive effect is supported by mechanistic evidence from both clinical and experimental studies 36, 40. Sesame seeds and sesame meal are rich in lignans, particularly sesamin and sesamolin, as well as vitamin E compounds such as gamma-tocopherol. These constituents have been shown to lower blood pressure through several biological pathways 40. Clinical trials in prehypertensive individuals indicate that black sesame meal supplementation can decrease systolic blood pressure and oxidative stress markers, while increasing plasma vitamin E concentrations 36. The antioxidant activity of sesame lignans and vitamin E likely contributes to improved endothelial function, enhanced nitric oxide bioavailability, and a reduction in lipid peroxidation, all of which are important in vascular health and blood pressure regulation 35, 41, 42. In addition to seeds, recent animal studies have highlighted the potent antihypertensive activity of sesame leaves. Key phytochemicals such as acteoside and pedaliin, which are present at high levels in the leaves, exhibit significant angiotensin I-converting enzyme (ACE) inhibitory activity 43. Inhibition of ACE can decrease the formation of angiotensin II, a major vasoconstrictor, thus leading to reduced peripheral resistance and lower blood pressure 43. Experimental evidence from spontaneously hypertensive rat models demonstrates that sesame leaf extract not only inhibits ACE activity *in vitro* but also significantly attenuates the progression of hypertension *in vivo* 42, 43. Together, these results support the potential of sesame supplementation as an adjunctive dietary strategy for blood pressure management, although further studies are needed to clarify the relative contributions of different sesame-derived compounds and their mechanisms of action in humans.

Regarding glycemic control, sesame supplementation exhibited beneficial effects on fasting blood glucose and HbA1c, reflecting potential improvements in insulin sensitivity and glucose metabolism. Previous experimental studies have attributed these beneficial effects to bioactive components such as sesamin, which may modulate glucose uptake and insulin signaling pathways in peripheral tissues 44, 45. However, despite these promising findings, sesame supplementation did not significantly affect fasting insulin levels or insulin resistance as measured by HOMA-IR, suggesting the observed glycemic improvements might involve mechanisms independent of direct insulin sensitivity enhancement 45. Further mechanistic investigations are needed to clarify these relationships.

Our analysis showed that sesame supplementation produced a moderate reduction in ALT, an important biomarker of liver injury and hepatic function. This result is consistent with preclinical and clinical evidence supporting the hepatoprotective properties of sesame-derived constituents, which may act through antioxidant and anti-inflammatory pathways 23. In a randomized, double-blind, controlled trial involving women with nonalcoholic fatty liver disease (NAFLD), daily intake of 30 grams of sesame oil for 12 weeks, combined with a hypocaloric diet, led to significant decreases in serum ALT and AST compared with sunflower oil. The study also reported improvements in hepatic steatosis grade, highlighting the potential of sesame oil to attenuate liver fat accumulation and improve liver health 23.

Sesamin, a lignan found in sesame, has been shown to protect against carbon tetrachloride-induced oxidative liver injury in rats by reducing serum liver enzyme levels and enhancing antioxidant enzyme activities. Another study demonstrated that sesamin administration mitigated acetaminophen-induced acute liver injury in mice, highlighting its role in attenuating oxidative stress and inflammatory responses 46, 47. Moreover, research indicates that sesamin can alleviate nonalcoholic steatohepatitis (NASH) by inhibiting hepatic pyroptosis, a form of programmed cell death associated with inflammation, thereby reducing liver enzyme levels and improving liver histology in high-fat diet-induced NASH models 48. These studies collectively suggest that sesame and its constituents, such as sesamin, possess hepatoprotective properties that may be beneficial in managing liver disorders like NAFLD and NASH.

Our results revealed no significant impact of sesame supplementation on body composition parameters, including body weight, BMI, body fat percentage, waist circumference, and hip circumference. The limited effect observed in these anthropometric measures suggests that sesame alone may be insufficient as a standalone intervention for weight management. The heterogeneous nature of body composition outcomes, combined with variations in dietary control, lifestyle factors, and baseline body composition among study populations, might partially explain these findings. Similarly, sesame did not exhibit significant effects on lipid profile parameters, including total cholesterol, triglycerides, LDL, and HDL cholesterol. Previous research has suggested that sesame lignans may have lipid-modulating properties by influencing cholesterol synthesis pathways or increasing hepatic cholesterol clearance 49, 50. The absence of significant findings in our meta-analysis may reflect substantial heterogeneity among studies in terms of sesame dosage, formulations, and participant baseline lipid levels. Future studies should investigate the impact of sesame supplementation stratified by baseline lipid profiles and varying sesame preparations to provide clearer insights.

Although sesame supplementation did not produce significant effects on inflammatory markers such as TNF-α, IL-6, and hs-CRP, this finding contrasts with preclinical evidence indicating that sesame compounds possess anti-inflammatory activity. Experimental studies have shown that constituents like sesamin may modulate inflammatory responses by suppressing key pro-inflammatory signaling pathways 51, 52. The lack of consistent effects observed in the present analysis could be attributed to limitations such as short intervention durations, suboptimal dosages, or the inclusion of participants with low baseline inflammation. To better assess the anti-inflammatory potential of sesame, future trials should target populations with elevated inflammatory markers and employ sufficient treatment durations and doses. The observed cardiometabolic benefits of sesame may be partially explained by its influence on molecular mechanisms involved in energy metabolism and inflammation. Sesamin has been reported to activate AMP-activated protein kinase (AMPK), which plays a central role in enhancing insulin sensitivity and reducing hepatic lipid accumulation by downregulating lipogenic pathways including SREBP-1c 50. In addition, it may promote the expression of peroxisome proliferator-activated receptor gamma (PPARγ) 53, a key regulator of glucose metabolism and adipocyte differentiation, potentially contributing to improved glycemic control. From an immunometabolic standpoint, sesamin has also been shown to suppress Toll-like receptor 4 (TLR4)-dependent activation of the NF-κB pathway 48, thereby reducing the production of pro-inflammatory cytokines. These mechanistic insights are consistent with the trends observed in our clinical findings and provide biological plausibility, although confirmation through larger and well-controlled human studies is still needed.

Notably, our analysis identified significant publication bias as indicated by Egger's regression, suggesting that studies with negative or inconclusive findings may be underrepresented in the current body of published literature. This bias can artificially inflate the perceived effectiveness of sesame supplementation by overemphasizing favorable outcomes, thereby limiting the validity and generalizability of the pooled effect estimates. Such distortion is particularly concerning in nutritional and complementary medicine research, where small sample sizes and selective reporting are common. The presence of publication bias underscores the need for cautious interpretation of our findings. Readers should be aware that the observed benefits of sesame may not fully reflect the true range of clinical effects, especially in real-world settings where study designs, dosages, and participant characteristics vary widely. It also highlights a broader systemic issue in evidence synthesis, where unpublished data—especially from industry-funded or underpowered trials—are often inaccessible or omitted from analysis. To address this issue, future research should prioritize the prospective registration of clinical trials, adherence to standardized reporting guidelines such as CONSORT, and the publication of all results regardless of statistical significance. Data-sharing initiatives and open-access repositories may also facilitate a more balanced and transparent evidence base. Ultimately, minimizing the influence of publication bias is essential for generating reliable conclusions and informing evidence-based dietary and clinical recommendations regarding sesame supplementation.

The observed antihypertensive and metabolic benefits of sesame supplementation in our analysis may have important implications for the prevention and management of retinopathy associated with cardiometabolic risk factors. Elevated blood pressure, impaired glycemic control, and increased oxidative stress are well-established contributors to the development and progression of retinopathy 54, particularly in individuals with diabetes and other cardiometabolic disorders 55, 56. By demonstrating that sesame supplementation can modestly lower systolic and diastolic blood pressure and improve metabolic markers, our findings suggest that incorporating sesame into dietary interventions could help mitigate microvascular complications affecting the retina. Furthermore, the antioxidant properties of sesame lignans and vitamin E may reduce oxidative damage to retinal vessels 36. Experimental research has also identified specific compounds in sesame leaves that inhibit angiotensin-converting enzyme (ACE) activity, which parallels the established protective role of ACE inhibitors in diabetic retinopathy 43, 57. ACE inhibition can attenuate vascular endothelial growth factor (VEGF) expression and help maintain the integrity of the retinal vascular barrier, thereby supporting the potential vascular protective effects of sesame leaf constituents 57. Taken together, these mechanisms support the possible role of sesame supplementation as an adjunctive nutritional approach for reducing the risk or slowing the progression of retinopathy among individuals with cardiometabolic risk factors.

Several limitations should be considered when interpreting the findings of this meta-analysis. First, the number of included randomized controlled trials (RCTs) was relatively small for certain outcomes, such as inflammatory markers and liver function enzymes, limiting the statistical power and generalizability of these specific results. The subgroup analyses, particularly those stratifying by intervention duration and sesame type, may be underpowered due to the limited number of studies available in each subgroup. Second, substantial heterogeneity was observed in several pooled estimates, including those related to lipid profiles and body composition outcomes. This heterogeneity likely stems from variations in study design, population characteristics, intervention duration, sesame formulations (e.g., seed, oil, extract, or sesamin), and dosage. Although we conducted subgroup analyses to explore potential sources of heterogeneity, not all confounding factors could be fully accounted for due to limited reporting in the original studies. Third, several studies included in the analysis lacked comprehensive reporting of blinding methods, allocation concealment, or adherence monitoring, which may have introduced bias. Although the Cochrane Risk of Bias assessment was used, some studies were judged as having “some concerns” in one or more domains, which may have influenced the pooled effect estimates. Fourth, the duration of most included trials was relatively short, with the majority lasting less than 12 weeks. This limits our ability to evaluate the long-term efficacy and safety of sesame supplementation, especially for chronic outcomes such as glycemic control, lipid modulation, and liver function. Fifth, publication bias was identified in certain outcomes, as suggested by Egger's regression and funnel plot asymmetry. This indicates the possible underreporting of studies with neutral or negative findings, potentially leading to an overestimation of sesame's beneficial effects. Lastly, while this meta-analysis aimed to comprehensively evaluate the cardiometabolic risk effects of sesame supplementation, the included studies varied in their control conditions. Some used placebo, others used alternative oils or active comparators, which may have influenced the results. Additionally, dietary background and lifestyle factors were often not controlled or reported, further limiting the interpretation of causality. Future well-designed RCTs with larger sample sizes, standardized sesame interventions, longer follow-up durations, and rigorous methodological quality are needed to confirm the observed findings and to better define the adjunctive therapeutic role of sesame in cardiometabolic health and its emerging application in retinopathy associated with metabolic risk factors.

## Conclusion

In conclusion, sesame supplementation may serve as a promising adjunctive strategy in the comprehensive management of cardiometabolic risk factors, including hypertension, glycemic control, and liver health. Additionally, its potential role in managing retinopathy associated with cardiometabolic disorders warrants further exploration. However, additional well-designed randomized controlled trials with standardized sesame formulations, optimal dosing, longer follow-up periods, and clearly defined patient populations are necessary to fully understand its therapeutic potential and the underlying mechanisms of its cardiometabolic effects.

## Figures and Tables

**Table 1:** Characteristics of included studies

Author (year) / Country	Diagnosis	Inclusioncriteria	Exclusion criteria	Sample size(% of male)/ age	Studydesign	Placebo using	Intervention/ Duration	Main Results	Secondary Results
~8 weeks									
Wu et al. (2009) / Australia	Cardiovascular disease risk in overweight and obese adults	1. BMI > 252. At least one metabolic syndrome risk factor or LDL > 3.4 mmol/L	BMI > 35, recent cardiovascular or cerebrovascular events (<6 months). Type I diabetes or insulin use. Chronic inflammation, arthritis, NSAIDs, vitamin E use. Lipid-lowering medications, Smoking, Alcohol > 40 g/day (men) or >30 g/day (women), and Abnormal liver/renal function.	N = 33 (55)/Mean age: 54.7 ± 8.6	RCT/single-blind/ crossover trial.	Iso-caloric placebo bar.	25 g/day sesame seed via breakfast bar (~50 mg/day sesame lignans)/ 5 weeks	No significant effects on: Blood lipids (total cholesterol, LDL, HDL, triglycerides), Blood pressure, Markers of inflammation (IL-6, TNF-α, hs-CRP), and Oxidative stress (F2-isoprostanes).	Significant increase (~8-fold) in urinary mammalian lignans (enterolactone, enterodiol) after sesame supplementation.
Miyawaki et al. (2009)/Japan	Mild hypertension	Mild hypertension (SBP: 137.3 ± 1.9 mmHg; DBP: 87.3 ± 1.2 mmHg), no secondary hypertension, no other diseases, and not using any medication.	Presence of secondary hypertension, use of any medications or supplements affecting blood pressure.	P: 13/ 47.3 ± 9.5 I: 12 / 51.0 ± 8.2	RCT/double-blind/placebo-controlled trial	Placebo capsules contained 180 mg of wheat germ oil	60 mg of sesamin daily (3 capsules twice daily; each capsule contained 10 mg sesamin + wheat germ oil)/ 4 weeks	1. Systolic BP decreased from 137.6 ± 2.2 to 134.1 ± 1.7 mmHg (↓3.5 mmHg, *p* = 0.044).2. Diastolic BP decreased from 87.7 ± 1.3 to 85.8 ± 1.0 mmHg (↓1.9 mmHg, *p* = 0.045).	1. No adverse events reported. 2. No significant change in BP during placebo periods.
Wichitsranoi et al. (2011)/ Thailand	Prehypertension (SBP: 120-139 mmHg or DBP: 80-89 mmHg)	Middle-aged adults (~50 years), prehypertension only (no other medical conditions), no use of medications or supplements affecting blood pressure, and not pregnant.	Any medication or supplement affecting blood pressure.	P: 15 (73)/50.3 ± 5.6 I: 15 (73) /49.3 ± 7.7	RCT/double-blind/placebo-controlled trial	Capsules with identical appearance and contents excluding black sesame meal.	2.52 g/day of black sesame meal (6 capsules/day, 0.42 g each)/ 4 weeks	1. SBP decreased significantly in sesame group (129.3 ± 6.8 → 121.0 ± 9.0 mmHg; *p* < 0.05).2. No significant BP change in placebo group.	No side effects reported.
Bahadoran et al. (2015)/ Iran	T2DM	1. Clinical diagnosis of type 2 diabetes mellitus for at least one year, no severe impairment of cardiac, hepatic, or renal function.2. Not receiving insulin injections or antioxidant supplements.	1. Severe impairment of cardiac, hepatic, or renal function.2. Pregnancy or lactation.3. Insulin therapy.4. Antioxidant supplementation use.	P: 16 (25)/52 ± 9 I: 20 (20) /50 ± 10	RCT/double-blind/Parallel-group design	Usual breakfast (without Tahini)	Replaced regular breakfast with 28 g/day Tahini (ground un-hulled sesame seeds)/ 6 weeks	1. Significant decrease (21.1%) in hs-CRP (inflammation marker; *p* < 0.05).2. Slight, non-significant improvements in fasting glucose, insulin, insulin sensitivity index, and HOMA-IR.	No adverse effects reported; compliance was good (>88%).
Helli et al. (2016)/ Iran	Rheumatoid Arthritis	1. Women with RA according to the 2010 American College of Rheumatology criteria.2. BMI: 25-35 kg/m².3. Stable pharmacological treatment (methotrexate, prednisone, sulfasalazine, hydroxychloroquine).	1. Pregnancy or lactation, smoking, use weight-loss medications.2. Use of antioxidants or vitamin supplements (in last 6 months).3. Participation in exercise/weight reduction programs.4. Presence of diabetes, cardiovascular disease, hypertension, infections, other inflammatory disorders, liver or kidney disorders.	P: 22/48.0 ± 9.8 I: 22/49.6 ± 9.7	RCT/double-blind/placebo-controlled clinical trial	Placebo capsules contained 200 mg starch	200 mg/day of sesamin/ 6 weeks	1. HDL-cholesterol significantly increased (*p*=0.007, compared to placebo). 2. No statistically significant differences between sesamin and placebo groups.	No adverse effects reported during the study.
Shahi et al. (2017)/ Iran	T2DM	1. Clinical diagnosis of Type 2 Diabetes Mellitus for >1 year.2. BMI <30 kg/m².	1. Severe cardiac, hepatic, thyroid, or renal impairment, pregnancy or lactation, and smoking.2. Use of insulin injections, multivitamins, antioxidants, omega-3, fiber supplements.3. Use of corticosteroids, NSAIDs, immunosuppressive drugs, or antibiotics.4. Participation in weight-loss or gain programs.	P: 22/51.72 ± 12.24 I: 22/50.00 ± 12.13	RCT/double-blind/placebo-controlled trial	Placebo capsules containing 200 mg/day starch	200 mg/day of sesamin /8 weeks	1. FBS (*p*=0.016 vs. placebo)2. HbA1c (*p*=0.002 vs. placebo)3. TNF-α (*p*=0.005 vs. placebo)4. No significant changes in insulin or HOMA-IR compared to placebo.	No adverse effects reported.
Farajbakhsh et al. (2019)/ Iran	Metabolic syndrome	Metabolic syndrome according to harmonized criteria (>3 of the following): Central obesity (waist circumference >102 cm for men, >88 cm for women), FBS >100 mg/dL, TG >150 mg/dL, HDL <40 mg/dL (men) or <50 mg/dL (women), and SBP >130 mmHg or Diastolic BP >85 mmHg.	1. Allergy/adverse reaction to sesame, sunflower oil, or vitamin E2. Thyroid, liver, kidney, autoimmune, or neoplastic diseases.3. Smoking or alcohol consumption.4. Use of antioxidants, vitamin/mineral supplements, NSAIDs, insulin, antihyperglycemic, antihypertensive, or lipid-lowering medications.5. Pregnancy or lactation.	P: 23 (41.9)/50.17 ± 7.6 I (sesame oil): 24 (30.2)/48.04 ± 7.67 I (sesame oil + vitamin E): 23 (35.0)/ 49.30 ± 10.03	RCT/single-blind/placebo-controlled trial (3 arms)	Sunflower oil	Group A: Sesame oil (30 mL/day) + vitamin E (400 mg/day)Group B: Sesame oil only (30 mL/day)Group C: Sunflower oil only (30 mL/day)/ 8 weeks	Sesame oil alone significantly improved (*p*<0.025 vs. baseline):↓ Total cholesterol, triglycerides, fasting blood glucose, HOMA-IR, systolic & diastolic BP, MDA.	1. No significant changes in anthropometric measures (BMI, weight, waist circumference) among groups.2. Good dietary adherence: no adverse events reported.
Ghoreishi et al. (2022)/ Iran	T2DM	1. FBS: 120-250 mg/dL2. Serum cholesterol: 210-250 mg/dL3. TG: >200 mg/dL4. Not using lipid- or glucose-lowering medications.	1. Insulin therapy, hypertension, cardiovascular, liver, kidney diseases, thyroid disorders.2. Previous sesame consumption.3. Pregnancy, lactation, use of oral contraceptives.4. Smoking, alcohol use, narcotic use.5. Change in diet or physical activity during the study.	P: 30 (36.7)/56.00 ± 6.11 I: 30 (33.3)/52.48 ± 5.72	RCT/open-label/parallel-group design	usual care	Sesame seeds: 60 grams/day/ 8 weeks	Significant reductions in sesame group compared to control:1. FBS: (*p* < 0.001)2. HbA1c: (*p* < 0.001)3. TC: (*p* < 0.001)4. TG:(*p* < 0.001)5. LDL-C: (*p* < 0.01)6. ALT enzymes: (*p* < 0.05)	1. No significant changes observed in AST enzyme.2. No adverse effects reported.
>8 weeks									
Aslam et al. (2019)/ Pakistan	T2DM	1. Clinically diagnosed Type 2 Diabetes Mellitus and participants not on insulin therapy.2. Stable medication dosage for at least 3 months prior to the study.3. BMI not exceeding 30 kg/m².	1. Insulin-dependent diabetes.2. Uncontrolled diabetes, coronary artery disease, renal diseases.3. Pregnancy or lactation.4. Planned changes in physical activity or dietary patterns.5. Use of dietary supplements or allergies to plant-derived foods.	P: 23 I: 23	RCT/double-blind/placebo-controlled clinical trial	Soybean oil as a placebo, identical appearance and quantity to sesame oil.	White Sesame Seed Oil: 30 mL/day consumed as part of regular diet/ 90 days	1. FBS (*p*< 0.05 vs. placebo )2. HbA1c (*p*< 0.05 vs. placebo)3. Insulin levels significantly increased (*p*< 0.05 vs. placebo)	1. Improved hepatic function markers (ALT, AST, ALP significantly reduced)2. Improved renal function markers (urea, creatinine, uric acid significantly reduced)
Vahedi et al. (2022)/Iran	NAFLD	1. Women aged 20-50 years and diagnosed NAFLD by ultrasonography.2. BMI between 25 and 40 kg/m².3. Regular consumption of sunflower oil as dietary oil before study.	1. Smoking, alcohol use, menopause, pregnancy, or lactation.2. Insulin therapy, use of hepatotoxic drugs, lipid-lowering medications, or drugs affecting liver enzymes.3. Hormone-dependent cysts, breast cancer history.4. Autoimmune diseases, renal failure, severe liver diseases, hereditary diseases.5. Recent special or weight-loss diets, multivitamin or omega-3 supplements.	P: 26/39.35 ± 5.89 I: 27/38.89 ± 6.91	RCT/double-blind/placebo-controlled clinical trial	Sunflower oil	1. Sesame Oil (SO): 30 g/day.2. Control: Sunflower oil: 30 g/day.3. All participants received a hypocaloric diet (-500 kcal/day)/ 12 weeks	Significant improvement with placebo after adjusting for confounders:1. ALT levels significantly reduced (*p*<0.05)2. AST levels significantly reduced (*p*<0.05)3. Fatty liver grade significantly improved (*p*<0.05)	No adverse effects were reported.

ALT: alanine transaminase; AST: aspartate aminotransferase; BMI: body mass index; DBP: diastolic blood pressure; FBS: fasting blood sugar; HbA1c: glycated Hemoglobin; HDL: high-density lipoprotein cholesterol; hs-CRP: high-sensitive C-reactive protein; LDL: low-density lipoprotein cholesterol; NAFLD: non-alcoholic fatty liver disease; P: placebo; I: intervention; SBP: systolic blood pressure; T2DM: type 2 diabetes mellitus; TC: total cholesterol; TG: triglycerides.

## References

[1] Triposkiadis F, Xanthopoulos A, Bargiota A, Kitai T, Katsiki N, Farmakis D (2021). Diabetes Mellitus and Heart Failure. J Clin Med.

[2] Ali MK, Pearson-Stuttard J, Selvin E, Gregg EW (2022). Interpreting global trends in type 2 diabetes complications and mortality. Diabetologia.

[3] Storz MA (2020). The Role of Vegan Diets in Lipotoxicity-induced Beta-cell Dysfunction in Type-2-Diabetes: A Narrative Review. J Popul Ther Clin Pharmacol.

[4] Macle L, Cairns JA, Andrade JG, Mitchell LB, Nattel S, Verma A (2015). The 2014 Atrial Fibrillation Guidelines Companion: A Practical Approach to the Use of the Canadian Cardiovascular Society Guidelines. Can J Cardiol.

[5] Danpanichkul P, Suparan K, Dutta P, Kaeosri C, Sukphutanan B, Pang Y (2024). Disparities in metabolic dysfunction-associated steatotic liver disease and cardiometabolic conditions in low and lower middle-income countries: a systematic analysis from the global burden of disease study 2019. Metabolism.

[6] Li CP, Chen CC, Hsiao Y, Kao CH, Chen CC, Yang HJ (2024). The Role of Lactobacillus plantarum in Reducing Obesity and Inflammation: A Meta-Analysis. Int J Mol Sci.

[7] Ramirez-Coronel AA, Ali Alhilali KA, Basheer Ahmed Y, Almalki SG, Karimian J (2023). Effect of sesame (Sesamum indicum L.) consumption on glycemic control in patients with type 2 diabetes: A systematic review and meta-analysis of randomized controlled trials. Phytother Res.

[8] Zhang J, Li L, Xiu F (2022). Sesamin suppresses high glucose-induced microglial inflammation in the retina i*n vitro* and *in vivo*. J Neurophysiol.

[9] Ahmad S, ElSherbiny NM, Jamal MS, Alzahrani FA, Haque R, Khan R (2016). Anti-inflammatory role of sesamin in STZ induced mice model of diabetic retinopathy. J Neuroimmunol.

[10] Demir S, Nawroth PP, Herzig S, Ekim Ustunel B (2021). Emerging Targets in Type 2 Diabetes and Diabetic Complications. Adv Sci (Weinh).

[12] Farajbakhsh A, Mazloomi SM, Mazidi M, Rezaie P, Akbarzadeh M, Ahmad SP (2019). Sesame oil and vitamin E co-administration may improve cardiometabolic risk factors in patients with metabolic syndrome: a randomized clinical trial. Eur J Clin Nutr.

[13] Mohammad Shahi M, Zakerzadeh M, Zakerkish M, Zarei M, Saki A (2017). Effect of Sesamin Supplementation on Glycemic Status, Inflammatory Markers, and Adiponectin Levels in Patients with Type 2 Diabetes Mellitus. J Diet Suppl.

[14] Helli B, Mowla K, Mohammadshahi M, Jalali MT (2016). Effect of Sesamin Supplementation on Cardiovascular Risk Factors in Women with Rheumatoid Arthritis. J Am Coll Nutr.

[15] Vahedi H, Atefi M, Entezari MH, Hassanzadeh A (2022). The effect of sesame oil consumption compared to sunflower oil on lipid profile, blood pressure, and anthropometric indices in women with non-alcoholic fatty liver disease: a randomized double-blind controlled trial. Trials.

[16] Wu JH, Hodgson JM, Puddey IB, Belski R, Burke V, Croft KD (2009). Sesame supplementation does not improve cardiovascular disease risk markers in overweight men and women. Nutr Metab Cardiovasc Dis.

[17] Higgins JPT, Cochrane Collaboration (2019). Cochrane handbook for systematic reviews of interventions. Second edition. ed. Hoboken, NJ: Wiley-Blackwell.

[18] Patti G, Chello M, Pasceri V, Colonna D, Carminati P, Covino E (2005). Dexamethasone-eluting stents and plasma concentrations of adhesion molecules in patients with unstable coronary syndromes: results of the historically controlled SESAME study. Clin Ther.

[19] Modesto-Filho (2008). ASFJ. Efeito do uso da farinha desengordurada do Sesamum indicum L nos níveis glicêmicos em diabéticas tipo 2. Revista Brasileira de Farmacognosia Brazilian Journal of Pharmacognosy.

[20] Ramezani-Jolfaie N, Aghaei S, Yazd EF, Moradi A, Mozaffari-Khosravi H, Zimorovat A (2020). Association of rs670 variant of APOA-1 gene with cardiometabolic markers after consuming sesame, canola and sesame-canola oils in adults with and without type 2 diabetes mellitus. Clin Nutr ESPEN.

[21] Fallah Z, Vasmehjani AA, Aghaei S, Amiri M, Raeisi-Dekordi H, Moghtaderi F (2024). Cardiometabolic risk factors are affected by interaction between FADS1 rs174556 variant and dietary vegetable oils in patients with diabetes: a randomized controlled trial. Sci Rep.

[22] Karatzi K, Stamatelopoulos K, Lykka M, Mantzouratou P, Skalidi S, Zakopoulos N (2013). Sesame oil consumption exerts a beneficial effect on endothelial function in hypertensive men. Eur J Prev Cardiol.

[23] Atefi M, Entezari MH, Vahedi H, Hassanzadeh A (2022). Sesame Oil Ameliorates Alanine Aminotransferase, Aspartate Aminotransferase, and Fatty Liver Grade in Women with Nonalcoholic Fatty Liver Disease Undergoing Low-Calorie Diet: A Randomized Double-Blind Controlled Trial. Int J Clin Pract.

[24] Amiri M, Raeisi-Dehkordi H, Moghtaderi F, Zimorovat A, Mohyadini M, Salehi-Abargouei A (2022). The effects of sesame, canola, and sesame-canola oils on cardiometabolic markers in patients with type 2 diabetes: a triple-blind three-way randomized crossover clinical trial. Eur J Nutr.

[25] Mirmiran P, Bahadoran Z, Golzarand M, Rajab A, Azizi F (2013). Ardeh (Sesamum indicum) could improve serum triglycerides and atherogenic lipid parameters in type 2 diabetic patients: a randomized clinical trial. Arch Iran Med.

[26] Moghtaderi F, Amiri M, Raeisi-Dehkordi H, Zimorovat A, Mohyadini M, Salehi-Abargouei A (2022). The effect of sesame, canola, and sesame-canola oils on cardiometabolic risk factors in overweight adults: a three-way randomized triple-blind crossover clinical trial. Phytother Res.

[27] Raeisi-Dehkordi H, Amiri M, Zimorovat A, Moghtaderi F, Zarei S, Forbes SC (2021). Canola oil compared with sesame and sesame-canola oil on glycaemic control and liver function in patients with type 2 diabetes: A three-way randomized triple-blind cross-over trial. Diabetes Metab Res Rev.

[28] Raeisi-Dehkordi H, Amiri M, Moghtaderi F, Zimorovat A, Rahmanian M, Mozaffari-Khosravi H (2021). Effects of sesame, canola and sesame-canola oils on body weight and composition in adults with type 2 diabetes mellitus: a randomized, triple-blind, cross-over clinical trial. J Sci Food Agric.

[29] Devarajan S, Singh R, Chatterjee B, Zhang B, Ali A (2016). A blend of sesame oil and rice bran oil lowers blood pressure and improves the lipid profile in mild-to-moderate hypertensive patients. J Clin Lipidol.

[30] Alipoor B, Haghighian MK, Sadat BE, Asghari M (2012). Effect of sesame seed on lipid profile and redox status in hyperlipidemic patients. Int J Food Sci Nutr.

[31] Devarajan S, Chatterjee B, Urata H, Zhang B, Ali A, Singh R (2016). A Blend of Sesame and Rice Bran Oils Lowers Hyperglycemia and Improves the Lipids. Am J Med.

[32] Sankar D, Rao MR, Sambandam G, Pugalendi KV (2006). Effect of sesame oil on diuretics or Beta-blockers in the modulation of blood pressure, anthropometry, lipid profile, and redox status. Yale J Biol Med.

[33] Sankar D, Rao MR, Sambandam G, Pugalendi KV (2006). A pilot study of open label sesame oil in hypertensive diabetics. J Med Food.

[34] Bahadoran Z (2015). A Sesame Seeds-Based Breakfast Could Attenuate Sub-Clinical Inflammation in Type 2 Diabetic Patients: A Randomized Controlled Trial. International Journal of Nutrition and Food Sciences.

[35] Miyawaki T, Aono H, Toyoda-Ono Y, Maeda H, Kiso Y, Moriyama K (2009). Antihypertensive effects of sesamin in humans. J Nutr Sci Vitaminol (Tokyo).

[36] Wichitsranoi J, Weerapreeyakul N, Boonsiri P, Settasatian C, Settasatian N, Komanasin N (2011). Antihypertensive and antioxidant effects of dietary black sesame meal in pre-hypertensive humans. Nutr J.

[37] Aslam F, Iqbal S, Nasir M, Anjum AA (2019). White Sesame Seed Oil Mitigates Blood Glucose Level, Reduces Oxidative Stress, and Improves Biomarkers of Hepatic and Renal Function in Participants with Type 2 Diabetes Mellitus. J Am Coll Nutr.

[38] Ghoreishi AS, Chatrnour G, Mahmoodi M (2022). The effect of sesame seeds on fast blood sugar, haemoglobin A1C, liver enzymes and lipid profile in patients with type 2 diabetes: a randomised clinical trial. Family Medicine & Primary Care Review.

[39] Sun Y, Ren J, Zhu S, Zhang Z, Guo Z, An J (2022). The Effects of Sesamin Supplementation on Obesity, Blood Pressure, and Lipid Profile: A Systematic Review and Meta-Analysis of Randomized Controlled Trials. Front Endocrinol (Lausanne).

[40] Khosravi-Boroujeni H, Nikbakht E, Natanelov E, Khalesi S (2017). Can sesame consumption improve blood pressure? A systematic review and meta-analysis of controlled trials. J Sci Food Agric.

[41] Lee CC, Chen PR, Lin S, Tsai SC, Wang BW, Chen WW (2004). Sesamin induces nitric oxide and decreases endothelin-1 production in HUVECs: possible implications for its antihypertensive effect. J Hypertens.

[42] Kong X, Li W, Guo LQ, Zhang JX, Chen XP, Liu WY (2015). Sesamin enhances nitric oxide bioactivity in aortas of spontaneously hypertensive rats. Ther Adv Cardiovasc Dis.

[43] Kitagawa T, Tashiro H, Uto T (2024). Antihypertensive and Angiotensin I-Converting Enzyme-Inhibitory Effects of the Leaves of Sesamum indicum and Bioactive Compounds. Chem Pharm Bull (Tokyo).

[44] Jalili C, Moradi S, Babaei A, Boozari B, Asbaghi O, Lazaridi AV (2020). Effects of Cynara scolymus L. on glycemic indices:A systematic review and meta-analysis of randomized clinical trials. Complement Ther Med.

[45] Mahdavi A, Moradi S, Askari G, Iraj B, Sathyapalan T, Guest PC (2021). Effect of Curcumin on Glycemic Control in Patients with Type 2 Diabetes: A Systematic Review of Randomized Clinical Trials. Adv Exp Med Biol.

[46] Lv D, Zhu CQ, Liu L (2015). Sesamin ameliorates oxidative liver injury induced by carbon tetrachloride in rat. Int J Clin Exp Pathol.

[47] Du H, Tong S, Kuang G, Gong X, Jiang N, Yang X (2023). Sesamin Protects against APAP-Induced Acute Liver Injury by Inhibiting Oxidative Stress and Inflammatory Response via Deactivation of HMGB1/TLR4/NFkappaB Signal in Mice. J Immunol Res.

[48] Zhang T, Zhou Y, Zhang Y, Wang DG, Lv QY, Wang W (2024). Sesamin ameliorates nonalcoholic steatohepatitis through inhibiting hepatocyte pyroptosis *in vivo* and *in vitro*. Front Pharmacol.

[49] Majdalawieh AF, Dalibalta S, Yousef SM (2020). Effects of sesamin on fatty acid and cholesterol metabolism, macrophage cholesterol homeostasis and serum lipid profile: A comprehensive review. Eur J Pharmacol.

[50] Liang YT, Chen J, Jiao R, Peng C, Zuo Y, Lei L (2015). Cholesterol-Lowering Activity of Sesamin Is Associated with Down-Regulation on Genes of Sterol Transporters Involved in Cholesterol Absorption. Journal of Agricultural and Food Chemistry.

[51] Kong P, Chen G, Jiang A, Wang Y, Song C, Zhuang J (2016). Sesamin inhibits IL-1β-stimulated inflammatory response in human osteoarthritis chondrocytes by activating Nrf2 signaling pathway. Oncotarget.

[52] Udomruk S, Kaewmool C, Pothacharoen P, Phitak T, Kongtawelert P (2018). Sesamin suppresses LPS-induced microglial activation via regulation of TLR4 expression. Journal of Functional Foods.

[53] Gina NNT, Kuo JL, Wu ML, Chuang SM (2024). Sesamin and sesamolin potentially inhibit adipogenesis through downregulating the peroxisome proliferator-activated receptor gamma protein expression and activity in 3T3-L1 cells. Nutr Res.

[54] Wondmeneh TG, Mohammed JA (2024). Prevalence of diabetic retinopathy and its associated risk factors among adults in Ethiopia: a systematic review and meta-analysis. Sci Rep.

[55] Peng X, Peng Y, Li L, Liu L, Zhou G, Cai Y (2025). Breakthroughs in diabetic retinopathy diagnosis and treatment using preclinical research models: current progress and future directions. Ann Med.

[56] Tomasoni M, Beyeler MJ, Vela SO, Mounier N, Porcu E, Corre T (2023). Genome-wide Association Studies of Retinal Vessel Tortuosity Identify Numerous Novel Loci Revealing Genes and Pathways Associated with Ocular and Cardiometabolic Diseases. Ophthalmol Sci.

[57] Gilbert RE, Kelly DJ, Cox AJ, Wilkinson-Berka JL, Rumble JR, Osicka T (2000). Angiotensin converting enzyme inhibition reduces retinal overexpression of vascular endothelial growth factor and hyperpermeability in experimental diabetes. Diabetologia.

